# Alphavirus Mutator Variants Present Host-Specific Defects and Attenuation in Mammalian and Insect Models

**DOI:** 10.1371/journal.ppat.1003877

**Published:** 2014-01-16

**Authors:** Kathryn Rozen-Gagnon, Kenneth A. Stapleford, Vanesa Mongelli, Hervé Blanc, Anna-Bella Failloux, Maria-Carla Saleh, Marco Vignuzzi

**Affiliations:** 1 Institut Pasteur, Viral Populations and Pathogenesis, CNRS UMR 3569, Paris, France; 2 University Paris Diderot, Sorbonne Paris Cite, Cellule Pasteur, Paris, France; 3 Institut Pasteur, Viruses and RNA Interference, UMR 3569, Paris, France; 4 Institut Pasteur, Arboviruses and Insect Vectors, Paris, France; New York State Department of Health, United States of America

## Abstract

Arboviruses cycle through both vertebrates and invertebrates, which requires them to adapt to disparate hosts while maintaining genetic integrity during genome replication. To study the genetic mechanisms and determinants of these processes, we use chikungunya virus (CHIKV), a re-emerging human pathogen transmitted by the *Aedes* mosquito. We previously isolated a high fidelity (or antimutator) polymerase variant, C483Y, which had decreased fitness in both mammalian and mosquito hosts, suggesting this residue may be a key molecular determinant. To further investigate effects of position 483 on RNA-dependent RNA-polymerase (RdRp) fidelity, we substituted every amino acid at this position. We isolated novel mutators with decreased replication fidelity and higher mutation frequencies, allowing us to examine the fitness of error-prone arbovirus variants. Although CHIKV mutators displayed no major replication defects in mammalian cell culture, they had reduced specific infectivity and were attenuated *in vivo*. Unexpectedly, mutator phenotypes were suppressed in mosquito cells and the variants exhibited significant defects in RNA synthesis. Consequently, these replication defects resulted in strong selection for reversion during infection of mosquitoes. Since residue 483 is conserved among alphaviruses, we examined the analogous mutations in Sindbis virus (SINV), which also reduced polymerase fidelity and generated replication defects in mosquito cells. However, replication defects were mosquito cell-specific and were not observed in *Drosophila* S2 cells, allowing us to evaluate the potential attenuation of mutators in insect models where pressure for reversion was absent. Indeed, the SINV mutator variant was attenuated in fruit flies. These findings confirm that residue 483 is a determinant regulating alphavirus polymerase fidelity and demonstrate proof of principle that arboviruses can be attenuated in mammalian and insect hosts by reducing fidelity.

## Author Summary

Chikungunya (CHIKV) is a re-emerging mosquito-borne virus that constitutes a major and growing human health burden. Like all RNA viruses, during viral replication CHIKV copies its genome using a polymerase that makes an average of one mistake per replication cycle. Therefore, a single virus generates millions of viral progeny that carry a multitude of distinct mutations in their genomes. In this study, we isolated CHIKV mutators (strains that make more errors than the wildtype virus), to study how higher mutation rates affect fitness in arthropod-borne viruses (arboviruses). CHIKV mutators have reduced virulence in mice and severe replication defects in *Aedes* mosquito cells. However, these replication defects result in selective pressure for reversion of mutators to a wildtype polymerase in mosquito hosts. To examine how mutators would behave in an insect model in absence of this genetic instability, we isolated mutators of a related virus, Sindbis virus (SINV). SINV mutators had no replication defect in fruit fly (*Drosophila*) cells, and a SINV mutator strain was stable and attenuated in fruit flies. This work shows proof of principle that arbovirus mutators can exhibit attenuation in both mammalian and insect hosts, and may remain a viable vaccine strategy.

## Introduction

During replication, RNA viruses generate approximately 1 error per 10^4^ nucleotides copied, giving rise to an immense population of genetically distinct but closely related variants [1], [2], [3], [4]. The genetic diversity of these “mutant swarms” is not detected by consensus sequencing, which to-date has been the basis for most studies of viral infection. However, this lack of information on genetic diversity has obscured crucial aspects of virus biology. Although RNA-dependent RNA polymerases (RdRp) have a high intrinsic error rate, their mutation rates can be altered to generate both higher and lower fidelity variants (antimutators and mutators, respectively) [5], [6], [7], [8], [9], [10], [11], [12], [13], [14]. Thus far, antimutator variants are thought to replicate more slowly, making fewer genomes with greater accuracy; in contrast, mutator variants have been shown to replicate more quickly, synthesizing more viral genomes but introducing many errors during the replication process [8], [15], [16], [17], [18]. Despite this, overall growth and titers of polymerase fidelity variants are not significantly different when grown in isolation in cell culture; for mutators the negative effects of accumulating deleterious mutations are only noticeable after several rounds of replication [6], [19]. In recent works, these variants have been useful in exploring how the course of viral infection is affected by either restricted or expanded population diversity [4], [10].

Current evidence indicates that mutation frequencies of RNA viruses have been optimized over time to be neither too accurate nor too erroneous [4], [6], [16], [20], [21], [22], [23]. It is thought that error-prone replication allows the virus to explore sequence space to gain adaptability and accumulate potentially advantageous mutations. For several RNA viruses, limiting viral population diversity has fitness costs *in vivo*. Despite similar *in vitro* growth phenotypes, variants that make fewer errors have reduced titers and exhibit restricted tropism in animal models [19], . This restriction in tropism may be due to cooperative inter-variant interactions or beneficial minority variants that are missing in a situation with restricted population diversity [19]. It is also proposed that high mutation rates of influenza A may contribute to altered tropism, allowing infection of new hosts [26]. Therefore, it seems that the relatively high error rates of RNA viruses generate a level of diversity that facilitates adaptive fitness advantages.

In contrast, there is also an upper threshold to mutation frequencies; if crossed, extreme error rates lead to the accumulation of deleterious mutations and loss of genetic integrity. Evidence for this is demonstrated by treatment of numerous RNA viruses with nucleoside analog mutagens, which increase mutation frequencies and result in extinction by lethal mutagenesis [27], [28], [29], [30], [31]. Although thus far RdRp mutators have not exhibited growth defects in isolation *in vitro*, a recent paper showed that HIV mutator and antimutator strains were less fit than wildtype in competition assays [32]. In addition, several studies recently report *in vivo* attenuation of mutator strains: Coxsackie virus B3 mutator strains present reduced viral titers in key organs and fail to establish persistent infections in mice [6], and a severe acute respiratory syndrome (SARS) coronavirus mutator strain exhibits reduced pathogenesis in several mouse models [7].

Antimutator and mutator variants are valuable tools to study where the threshold of advantageous polymerase error exists for viruses facing different selective pressures. In this respect, arboviruses represent a special evolutionary position due to their need to replicate in disparate hosts, which is accompanied by distinct selective pressures. Arbovirus fitness is not necessarily reduced due to obligate host-cycling (alternating passages of CHIKV did not limit viral fitness), yet it has been shown that evolvability may be reduced due to these evolutionary constraints [33], [34], [35], [36], [37], [38]. For alphaviruses, evidence suggests that viral diversity is most restricted in the insect host, due to more stringent population bottlenecks and selective pressures [33], [34], [35], [37], [39], [40]. Since minority variants are thought to play important roles in arbovirus pathogenesis, transmission, and emergence [25], [41], [42], [43], [44], [45], the implications of altered polymerase fidelity and mutation rates merit further study. Recently, this question was partially addressed using a chikungunya virus antimutator variant [25].

Chikungunya virus (CHIKV) is a re-emerging arbovirus, transmitted by *Aedes* species mosquitoes. This positive-stranded RNA virus (family *Togaviridae*, genus *Alphavirus*) has an 11.8 kB genome, of which the first 7.5 kb encode four nonstructural proteins (nsP1-4) involved in diverse processes including RNA synthesis, immune evasion, and host tropism [46], [47], [48], [49], [50], [51], [52]. In most cases, functions of these proteins are putative in CHIKV and have only been shown in related model viruses, such as Semliki forest virus and Sindbis virus [53], [54], [55], [56]. Nsp4 is the RdRp, responsible for nucleotide incorporation during replication [57]. Previously, we isolated an antimutator strain of CHIKV by passaging virus in ribavirin, an RNA nucleoside analog. Ribavirin causes nucleotide misincorporation by the RdRp, adding selective pressure for an intrinsically more faithful polymerase [14], [58], [59]. This antimutator strain harbored a single amino acid change (483Y) in nsp4. Although 483Y showed no growth defects *in vitro*, the variant was moderately attenuated *in vivo* in both mammalian and mosquito hosts [25]. However, no arbovirus mutators have been isolated thus far. To this end, we mutated the conserved cysteine residue at position 483 to obtain several mutators in the arboviruses CHIKV and SINV, confirming this position's importance in determining alphavirus fidelity. We used these novel mutator strains to examine how increased polymerase error affects arbovirus fitness *in vitro* and *in vivo*; interestingly, mutator strains presented distinct cell- and host-specific phenotypes.

## Materials and Methods

### Cells and viruses

Mammalian cell lines Vero, HeLa, and BHK-21 were maintained in DMEM (Gibco) supplemented with 10% newborn calf serum (NCS, Gibco) and 1% penicillin-streptomycin (P/S, Sigma), at 37°C with 5% CO_2_. Mosquito cell lines C6/36 and U4.4 (*Aedes albopictus*) and Aag2 (*Aedes aegypti*) were grown in L-15 media, supplemented with 10% fetal bovine serum (FBS, Gibco), 1% P/S, 1% tryptose phosphate, and 1% non-essential amino acids (NEAA), at 28°C with 5% CO_2_. *Drosophila melanogaster* S2 cells were grown in Schneider's *Drosophila* media (Gibco), supplemented with 10% FBS, 1% L-Glutamine, and 1% P/S at 25°C.

Wildtype CHIKV was generated from the La Reunion strain 06-049 infectious clone, previously described [33]. Nsp4 position 483 mutants were generated by site-directed mutagenesis of the infectious clone using the QuikChange II XL Site-Directed Mutagenesis kit (Stratagene). All newly generated DNA plasmids were Sanger sequenced in full (GATC Biotech) to confirm mutagenesis of position 483 and to ensure no second-site mutations were introduced. Select SINV mutants were constructed in the same fashion from the pTR339 wildtype infectious clone [60]. CHIKV and SINV expression plasmids were linearized with *Not*I or *Xho*I respectively, purified by phenol-chloroform extraction and ethanol precipitation, and subsequently used for *in vitro* transcription of viral RNAs using the SP6 mMESSAGE mMACHINE kit (Ambion). RNAs were then purified by phenol:chloroform extraction and ethanol precipitation, quantified, diluted to 1 µg/µl and stored at −80°C.

For RNA transfections, BHK-21 cells were trypsinized, washed twice with ice-cold PBS, and resuspended at a concentration of 2×10^7^ cells/ml in ice-cold PBS. Cells (0.390 ml) were mixed with 10 µg of *in vitro* transcribed viral RNA, placed in 2 mm cuvette and electroporated at 1.2 kV, 25 µF with infinite Ω in a XCell Gene Pulser (BioRad). Cells were allowed to recover for 10 minutes at room temperature then mixed with 6 ml of pre-warmed media and placed into a T-25 flask. After 48 hours incubation at 37°C, viral titers were determined by standard plaque assay. In brief, 10-fold serial dilutions of each virus in DMEM were incubated on a confluent monolayer of Vero cells for 1 hour at 37°C. Following incubation, cells were overlaid with 0.8% agarose dissolved in DMEM and 2% NCS and incubated at 37°C for 72 hours. The cells were then fixed with 4% formalin for 1 hour, the agarose plugs were removed, and plaques were visualized by the addition of crystal violet. Plaque size was quantified by scanning the crystal violet-stained cell monolayer, then quantifying the size of each individual plaque in square millimeters using ImageJ (http://rsbweb.nih.gov/ij).

Each virus was then passaged once over a 70–80% confluent monolayer of BHK-21 cells, titered as described above, aliquoted, and stored at −80°C until use. To analyze each virus for reversion at position 483, viral RNA was extracted for each electroporation and BHK-21 passage using TRIzol reagent (Invitrogen). For CHIKV, this RNA was used to amplify a 3184 bp region corresponding to nucleotides (4522–7706), which included position 483, using the forward primer (5′-GATGAGCACATCTCCATAG-3′) and the reverse primer (5′-GTTTGGGTTGGGATGAACT-3′) and the Titan One Tube RT-PCR Kit (Roche). For SINV, a 2225 bp region (nucleotides 6556–8781) was amplified in the same fashion using forward primer (5′-ACCAGGCACGAAACACACAGAA-3′) and reverse primer (5′-ACTGGGCGGAAGTCTGTATGCG -3′). Each PCR product was cleaned using the Nucleospin PCR and Gel Extraction Kit (Macherey-Nagel) and Sanger sequenced at position 483/482 to confirm genetic stability. At passage 3, all viruses used were fully sequenced to ensure no second site mutations.

### Ribavirin sensitivity assay

HeLa cells (250,000 cells/well in 12-well tissue culture plates) were pre-treated for two hours with either media containing no mutagen, or media containing 200 µM or 400 µM ribavirin (Sigma). Post-treatment, media was removed and the cells were inoculated with virus in DMEM at an MOI 0.1 for one hour at 37°C. Following incubation, mutagen-containing media was replaced and cells were incubated for 72 hours at 37°C. Virus was harvested at 72 hours and mean titers were obtained by TCID_50_. In brief, a 96-well tissue culture plates was plated for each virus with 1×10^4^ Vero cells/well. Viruses were serially diluted in 8 ten-fold dilutions in DMEM. Each dilution was distributed in a row of the 96-well plate, with each well receiving 100 µl of diluted virus. Viruses and cells were incubated 5–7 days at 37°C with 5% CO_2_. Following incubation, cells were fixed with 50 µl of 4% formalin for 30 minutes. All media were removed, and 50 µl of crystal violet was added to each well. Viruses that exhibited significant sensitivity or resistance compared to wildtype at P<0.05 or greater at either 200 µM or 400 µM ribavirin were considered potential fidelity variants, and mutation frequencies were estimated (Table 1).

**Table 1:** Characterization of CHIKV 483 polymerase variants.

Mutation	Genetic Stability	Hydrophobicity^a^	Plaque Size^b^	Ribavirin Sensitivity^c^	Mutation Frequency^d^
Cys (WT)	Stable	0.04	1.686	N/A	4.3
C483I	Stable	0.73	2.038	WT	ND
C483F	Stable	0.61	2.319	WT	ND
C483V	Stable	0.61	1.348	WT	ND
C483L	Stable	0.53	0.387*	WT	ND
C483W	Stable	0.37	1.072*	Sensitive	6.4**
C483M	Stable	0.26	2.032	Resistant	4.9
C483A	Stable	0.25	0.785*	Sensitive	6.0*
C483G	Stable	0.16	0.513*	Sensitive	8.3***
C483Y	Stable	0.02	2.083	Resistant	4.1
C483P	Not recovered	−0.07	ND	ND	ND
C483T	Stable	−0.18	0.532*	Sensitive	5.0
C483H	Reverted to Y	−0.4	ND	ND	ND
C483E	Reverted to V	−0.62	ND	ND	ND
C483N	Stable	−0.64	0.771*	Resistant	3.8
C483Q	Stable	−0.69	0.169*	Sensitive	4.6
C483D	Reverted to Y	−0.72	ND	ND	ND
C483K	Reverted to T	−1.1	ND	ND	ND
C483R	Reverted to L	−1.8	ND	ND	ND
C483S	Not recovered	−0.26	ND	ND	ND

^a^ Hydrophobicities from Eisenberg et al., 1982 [94].

^b^ Unit of measurement is mm^2^, * P-value<0.01, Student's *t*-test.

^c^ Determined in HeLa cells; resistance or sensitivity is noted where percent survival is significantly different from WT at either concentration tested.

^d^ per 10,000 nucleotides, *P<0.05, **P<0.01, ***P<0.001, χ^2^ test.

ND, not determined.

### Mutation frequencies by molecular cloning

To determine mutation frequencies, all mutants were electroporated in tandem into BHK-21 cells. Supernatants were collected 48 hours later and viral RNA was extracted. For CHIKV, an approximately 800 bp region corresponding to nucleotides 9943–10726 was amplified of the E1 region of the genome using forward primer 5′-TACGAACACGTAACAGTGATCC-3′ and reverse primer 5′-CGCTCTTACCGGGTTTGTTG-3′. For SINV, the analogous region was amplified using forward primer 5′-TACGAACATGCGACCACTGTTC-3′ and reverse primer 5′-CGCTCGGAGCGGATTTACTG-3′, and approximately 500 bases of this fragment was included in the analysis. Amplified fragments were purified as described above, and 3 µl of each product was modified by a 3′ A-overhang addition reaction (1 µl AmpliTaq Gold 10× buffer, 1 µl 10 mM dATP). Modified products were cloned using the TopoTA cloning kit (Invitrogen), and single colonies were picked for sequencing. Mutation frequencies were determined as previously described [61]. Mutation frequencies in mosquito cells were obtained in the same fashion using the samples obtained from C6/36 growth curves (we determined mutation frequency for a wildtype sample electroporated into C6/36 cells, and there was no difference between samples generated by infection or electroporation; the nonviability of mutators transfected into mosquito cells made it impossible to estimate mutation frequencies in C6/36 by electroporation). We sequenced approximately 75 clones per viral population in C6/36 cells. Mutation frequencies from mouse muscle were determined using RNA extracted from homogenized muscle samples from mice that most closely represented the median titer for that variants. For estimating *in vivo* mutation frequencies, a minimum of 50 clones were sampled per population. To confirm that the presence of RNA or aberrant viral particles in supernatants/homogenates did not affect mutation frequencies, we purified virus on 20% sucrose cushion and re-estimated mutation frequencies; no differences were observed.

### Population diversity by deep sequencing

To estimate the population diversity of variants by deep sequencing, cDNA libraries were prepared by Superscript III from RNA extracted from virus generated in BHK-21 or C6/36 cells, and the viral genome was amplified using a high fidelity polymerase (Phusion) to generate 5 overlapping amplicons 2–3 kb in length. PCRs were fragmented (Fragmentase), multiplexed, clustered, sequenced in the same lane with Illumina cBot and GAIIX technology and analyzed with established deep sequencing data analysis tools and in house scripts. Briefly, per-base Phred quality scores were utilized to trim bases with error probabilities higher than 0.001, and sequences with less than 16 bases after trimming were discarded. For this purpose we used the fastq-mcf tool from the ea-utils toolkit at http://code.google.com/p/ea-utils [62]. The alignment step is performed using Burrows Wheeler Aligner [63] and Pileup is performed using SAMtools [64]. Once the pileup is done, an in-house script collects the data per-position and calculates the variance at each nucleotide position by root mean square deviation (RMSD) and determines the mean variance and standard error across the whole genome [65].

### Neutralization assays

To estimate population diversity in a phenotypic assay, we performed neutralization assays using viruses which had been passaged 3 times on BHK-21 cells, using the n Neutralizing antibody CHK-102 (a kind gift from Dr. M.S. Diamond [66]). 100 pfu of wildtype and mutator CHIKV strains were incubated for 1 hour at 37°C with serial dilutions of antibody, ranging from 2 µg/ml to .0001 µg/mL, or left untreated. Virus-antibody complexes were added to pre-seeded confluent monolayers of Vero cells, and allowed to bind at 37°C for 1 hour. Assays were then overlayed with agarose and developed as described above for a plaque assay. Plaques were counted and normalized to the untreated control for each virus.

### Viral replication and extracellular RNA synthesis

Virus growth was evaluated for WT and all mutant viruses in BHK-21, C6/36, U4.4, Aag2, and S2 cells and titers were determined by TCID_50_ on Vero cells as described above. Using the 24 hour time point from the C6/36 growth curve, we also performed a cytopathic effect (CPE) assay on C6/36 cells on all viruses (CellTiter 96 AQueuos One Solution Cell Proliferation Assay (MTS) kit; Promega). We obtained similar titers by standard TCID_50_ and CPE assay, indicating that viruses amplified on mosquito cells were still equally infectious when titered on Vero cells. For CHIKV, genome copy number was determined by extracting viral RNA from the supernatant at each time point using the TRIzol reagent and performing quantitative RT-PCR (qRT-PCR) using the TaqMan RNA-to-Ct kit (Applied Biosystems). Ct values were determined in duplicate based on amplification of nsp4 transcripts using forward (5′-TCACTCCCTGCTGGACTTGATAGA-3′) and reverse (5′-TGACGAACAGAGTTAGGAACATACC-3′) primers and probe 5′- [6-FAM] AGGTACGCGCTTCAAGTTCGGCG-3′ as previously published [33], [67]. To determine genome copy number for SINV, viral RNA was extracted in the same manner and quantitative PCR was performed based on amplification of nsp3 transcripts using forward (5′-AAAACGCCTACCATGCAGTG-3′) and reverse (5′-TTTTCCGGCTGCGTAAATGC-3′) primers and the SYBR green PCR master mix (Applied Biosystems). Standard curves were performed in each run using samples of *in vitro* transcribed CHIKV or SINV RNA.

### Northern blot analysis

*In vitro* transcribed RNA was transfected in BHK-21 cells in duplicate, as described above or at 28°C, including RNA from a construct in which the polymerase active site (GDD) was replaced with GNN by site-directed mutagenesis to abrogate replication and alongside a mock transfection where no RNA was added. Transfections in C6/36 and U4.4 cells were modified by pulsing with 250 V, 50 µF, and 550 Ω. Forty-eight hours post-transfection, supernatant containing progeny virus was collected. Cells were washed twice in PBS and RNA was TRIzol (Invitrogen) extracted, quantified and diluted to the same concentration. Samples were prepared in NorthernMax formaldehyde loading dye (Ambion) with 1 µl of ethidium bromide, heated to 65°C for 10 minutes, then separated on a 1.2% LE agarose (Lonza) gel containing 1× morpholinepropanesulfonic acid (MOPS) running buffer (Ambion) and 6.7% formaldehyde. RNA was transferred onto nitrocellulose membrane, cross-linked by ultraviolet irradiation (UVP), and prehybridized at 68°C for 1 hour in ULTRAhyb ultrasensitive hybridization buffer (Ambion). A plasmid used for the expression of CHIKV RNA probes corresponding to the 3′ portion of the E2 glycoprotein was generated by first amplifying the region of the CHIKV genome from 8703 (5′-GAAGCGACAGACGGGACG-3′) to 9266 (5′-GTTACATTTGCCAGCGGAA-3′) by PCR and subsequently TOPO-TA cloning the PCR product into the pCRTOPO-II vector. RNA probes complementary to positive strand RNA were labeled with ^32^P using the MAXIscript SP6 *In Vitro* Transcription Kit (Ambion), unincorporated nucleotides were removed using illustra MicroSpin S200 HR columns (GE healthcare), and probe was hybridized to the membrane overnight at 68°C. Membranes were washed several times at 68°C with 0.1× SSC with 0.1% SDS, then imaged using Amersham Hyperfilm MP autoradiography film (GE Healthcare). Quantification was done using ImageJ (http://rsbweb.nih.gov/ij).

### Mouse infections

C57BL/6 mice (Janvier) or CD-1 mice (Charles River) were housed according to Institut Pasteur guidelines in biosafety level 3 isolators, with the approved experimental protocol #10.620, reviewed by the Institut Pasteur ethics committee under dossier #CETEA 2013-0021. At 8-days old, litters of C57BL/6 were inoculated with 200 pfu of wildtype or mutant CHIKV viruses subcutaneously (n = 4/variant). Eight-day old CD-1 litters were inoculated with 100 pfu of wildtype or mutant SINV strains in the same fashion, and monitored for symptoms of hind limb paralysis and survival. In addition, seven days post-infection, CHIKV and SINV-infected mice were sacrificed and brains, thigh muscles, livers and blood were harvested and homogenized in 300 µl of PBS at 30 shakes/second for 2 min (MM300 Retsch). RNA was extracted and viral genome copies were determined by qRT-PCR as described.

### Mosquito infections

Principal CHIKV vectors *Ae. albopictus* Providence (ALPROV, F8 generation) from La Reunion and *Ae. aegypti* Paea (PAE, a lab colony at Institut Pasteur since 1994) from Tahiti, in French Polynesia were fed on artificial bloodmeals containing 10^6^ pfu/ml of virus in PBS-washed rabbit blood [68]. CHIKV wildtype and mutators were fed to both *Ae. albopictus* and *Ae. aegypti*, and SINV wildtype and mutator 482G were fed to *Ae. aegypti*. The blood meals were warmed to 37°C and presented to 10 day-old females in membrane feeders, and engorged mosquitoes were incubated for 7 days. Seven days post infection, mosquitoes were dissected to obtain legs and wings, and saliva was obtained by *in vitro* transmission assay; in brief, mosquitoes were salivated for 30–45 min by placing the proboscis in a pipette tip containing FBS. Following salivation, bodies were frozen. To confirm ingestion, a sample of engorged mosquitoes was immediately homogenized at time 0. Samples were homogenized as described for mouse tissues, RNA was extracted, and qRT-PCR was performed. A standard curve was generated using serial dilutions of a CHIKV bloodmeal of known titer.

### Fruit fly infections

*Drosophila melanogaster* flies (strain w*^1118^*) were reared on standard medium at 25°C. Three- to four-day-old female flies were injected with 50 nL of a virus dilution containing 400 pfu in 10 mM Tris-HCl (pH 7.5) using a Drummond nanoject injector as previously described [69]. Fly mortality at day 1 was attributed to damage produced by the injection, and these flies were excluded from further analyses. Mortality was monitored daily for 10 days, and every 3–4 days flies were transferred to fresh vials. In all experiments, 30–60 flies per genotype group were injected. Homogenates of individual flies were titrated on by plaque assay on Vero cells, as described above.

### Statistical tests

All experiments were performed in triplicate unless noted otherwise. Statistics, noted where applied, were performed in Microsoft Excel and GraphPad Prism. P-values>0.05 were considered non-significant (ns).

## Results

### Characterization of CHIKV polymerase variants

We previously described a CHIKV antimutator variant that possessed a single amino acid change from a cysteine to a tyrosine at position 483 (C483Y) of the RNA-dependent RNA polymerase nsp4 [25]. Since Coxsackie virus B3 mutator strains are situated in a structurally analogous area, we hypothesized that this position plays important roles in modulating intrinsic CHIKV RdRp fidelity [6]. To address this, we substituted each amino acid at position 483 of the CHIKV full-length infectious clone (Table 1). After three passages in BHK-21 cells, viruses were Sanger sequenced to determine genetic stability. Of the 19 substitutions, 12 were viable and genetically stable (Table 1). This high number of viable variants indicates that position 483 has structural plasticity and can tolerate a wider range of substitutions than in previous attempts at generating fidelity variants of other RNA viruses [6], [19]. Interestingly, unstable viruses did not readily revert to wildtype, but mutated to other variants, including the antimutator form of the protein, 483Y (Table 1). The only strict biochemical requirement we observed was a necessity for uncharged residues, as all variants with charged residues (483D, E, H, K, or R) were unstable or not recoverable. In addition, we observed a general correlation between hydrophobicity of the substituted amino acid and stability or viability of the variant, where hydrophobic amino acids were preferred. Finally, as a first characterization of virus fitness, we measured the mean size of plaques. Variants 483A, G, L, N, Q, T, and W had significantly smaller plaques than wildtype (Table 1).

### Identification of ribavirin sensitive variants

**Figure 1:**
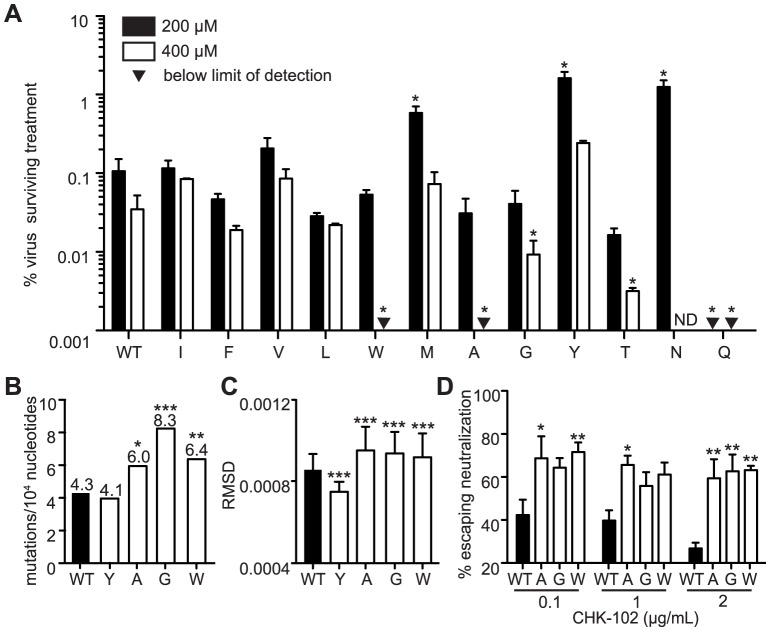
Mutagenizing position 483 variants allows isolation of mutator variants. (A) HeLa cells, treated with 200 µM or 400 µM ribavirin, or left untreated, were infected at an MOI of 0.1. The percentage of infectious progeny virus surviving treatment at both concentrations of ribavirin relative to the untreated control is shown (mean values ± SEM, n = 3, *P at least<0.05, two-way ANOVA with Bonferroni posttest). ND, not determined. (B) Average mutation frequencies of WT CHIKV and variants with significantly altered fidelity. Mutation frequency is shown as the mean number of mutations per 10,000 nucleotides sequenced by molecular cloning. Variants 483A, G and W made significantly more errors than WT virus (*P<0.05, **P<0.01, ***P<0.001, χ^2^ test). (C) Average diversity of confirmed fidelity variants at each position across the genome. The root-mean-square-deviation (RMSD) is shown (mean values ± SEM, ***P<0.001, Mann-Whitney *u* test). (D) Neutralization assay showing enhanced escape of mutators due to greater population diversity (mean values ± SEM, *P<0.05, **P<0.01, ***P<0.001, two-way ANOVA with Bonferroni posttest).

Because polymerase fidelity variants have altered intrinsic rates of (in)correct nucleotide incorporation, they have often been identified by their relative resistance or sensitivity to nucleoside analog RNA mutagens [14], [19], [25]. Therefore, we addressed the sensitivity of all 12 genetically stable variants to ribavirin (Table 1 and Figure 1A). Viruses were grown in the presence of either 200 µM or 400 µM ribavirin, or left untreated. We expect antimutator variants (such as 483Y) to demonstrate resistance, and mutator variants to demonstrate sensitivity when compared to wildtype. As previously described, the antimutator 483Y demonstrated significantly higher survival than wildtype (P<0.001, two-way ANOVA) as did 483M and 483N (P<0.001 for both, two-way ANOVA). Additionally, we identified several mutator candidates that were significantly more sensitive to ribavirin (483A, G, W, T, Q; P<0.05 for all, two-way ANOVA). All ribavirin-sensitive variants presented small-plaque phentoypes, as well as variant 483N (P<0.01 for all, Student's *t*-test). Though these variants presented small plaque phenotypes, virus stocks reached wildtype-like titers, with the exception of 483N and 483Q (Table 1).

### Confirmation of mutator phenotypes by molecular clone sequencing

As observed previously for picornaviruses [5], [6], [14], the ribavirin-resistant and -sensitive phenotypes of these CHIKV variants suggested altered polymerase fidelity. To address this further in a genetic assay, we estimated the mutation frequencies of each variant that demonstrated significantly altered ribavirin sensitivity at either concentration of ribavirin. Viral RNA from the supernatants of BHK-21 cells was extracted, and an approximately 800 nucleotide fragment of the E1 genome was amplified by RT-PCR and TOPO cloned as previously described [61]. We sequenced approximately 150 individual clones per viral population (corresponding to an average of 122,200 nucleotides) to calculate the mutation frequencies (Figure 1B and Table 1). Since previous studies with 483Y required >350 clones per population to distinguish more subtle differences in mutation frequencies [25], we could only statistically confirm the altered fidelities of three mutator strains (483A, G and W; P<0.05, P<0.001, P<0.01, respectively, χ^2^ test) (Figure 1B). We excluded variants that did not exhibit significant fidelity differences compared to wildtype (483M, N, and Q). As a complementary approach, we performed deep sequencing on these same virus populations to characterize the relative diversity in these virus populations. In accordance with the mutation frequency data, the mean variance across the whole genome was significantly lower for the antimutator 483Y variant (P = 0.0006, Mann-Whitney *u* test) and significantly higher for the 483A, G and W mutator variants, compared to wildtype virus (P<0.0001 for all, Mann-Whitney *u* test; Figure 1C).

### Chikungunya mutator strains have normal replication and reduced specific infectivity in mammalian cells

**Figure 2:**
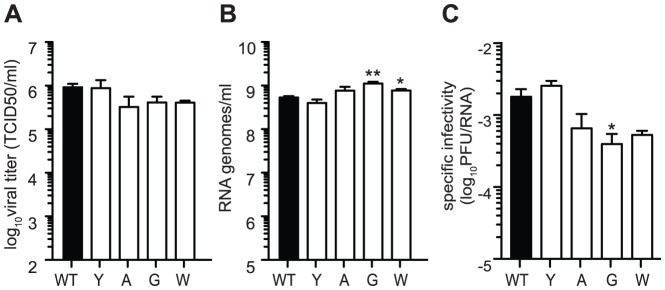
Mutators produce more RNA genomes and have lower specific infectivity than wildtype. Production of infectious particles determined by TCID_50_ (A) and genome copies determined by qRT-PCR (B) were measured in BHK-21 cells. No significant difference compared to WT was found for any variant in terms of titer. Mutators 483A, G, W tended to have greater amounts of extracellular RNA genomes at 24 hours (mean values ± SEM, n = 3, *P<0.05, **P<0.01, two-way ANOVA with Bonferroni posttest). (C) Specific infectivity (PFU/RNA genomes from A and B) of mutator strains were lower than that of WT virus (mean values ± SEM, n = 3, *P<0.05, two-way ANOVA with Bonferroni posttest).

Next, we examined growth of these variants in mammalian cells. As seen previously, the antimutator 483Y presented no significant difference in amount of progeny virus (Figure 2A) or number of genome copies (Figure 2B). As observed with Coxsackie virus mutators, CHIKV mutator strains (483A, G, and W) generated the same or more genomes than wildtype virus (Figure 2B), but slightly fewer infectious progeny (Figure 2A). Consequently, these mutator variants have a lower specific infectivity than wildtype in mammalian cells (Figure 1C). This is consistent with previously published results showing that mutator variants make more lethally mutagenized RNA [6], [22], [28], [70].

### Mutator strains of chikungunya virus are attenuated in mice

**Figure 3:**
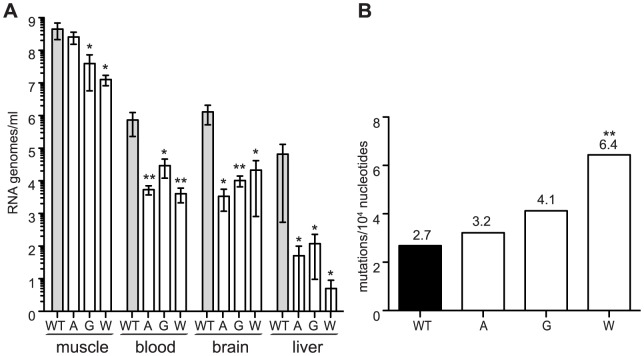
Mutator variants 483A, G, and W are attenuated in mice. (A) Seven days post-infection, mutators have significantly fewer RNA genomes present in the muscle, blood, brain, and liver of newborn mice compared to WT (median values ± IQR are shown, n = 4, *P<0.05, **P<0.01, two-way ANOVA with Bonferroni posttest). (B) Average mutation frequencies of WT CHIKV and mutators 483A, G and W from the muscle of a mouse representing the median titer of each group. All mutators retain an elevated mutation frequency, with the most attenuated variant (C483W) making significantly more errors than WT virus (**P<0.01, χ^2^ test).

Recently, low fidelity polymerase mutators of Coxsackie virus and exonuclease activity deficient mutators of coronaviruses were shown to be attenuated in mice [6], [7]. To determine whether this holds true for alphaviruses, we administered a sublethal infection of either wildtype or 483A, G and W viruses to 8-day old C57BL/6 mice. At 7 days of infection, when titers peak and virus is rapidly cleared thereafter, viral loads were determined in different compartments (muscle, blood, brain, liver). Viral loads were significantly lower for all three mutator strains in each tissue (Figure 3A). Since the *in vivo* mutation frequencies of mutator strains had not been previously reported, we examined the virus populations in the muscle of the wildtype- or the mutator-infected mouse that presented the median viral load. Although we cannot predict whether selection will act differently on these variants in mice to potentially skew the mutation frequencies, they remained elevated to varying degrees for the mutator strains. Interestingly, higher mutation frequencies *in vivo* correlated with increased attenuation (Figure 3B).

### Chikungunya mutator variants present a host-specific replication defect in mosquito cells

**Figure 4:**
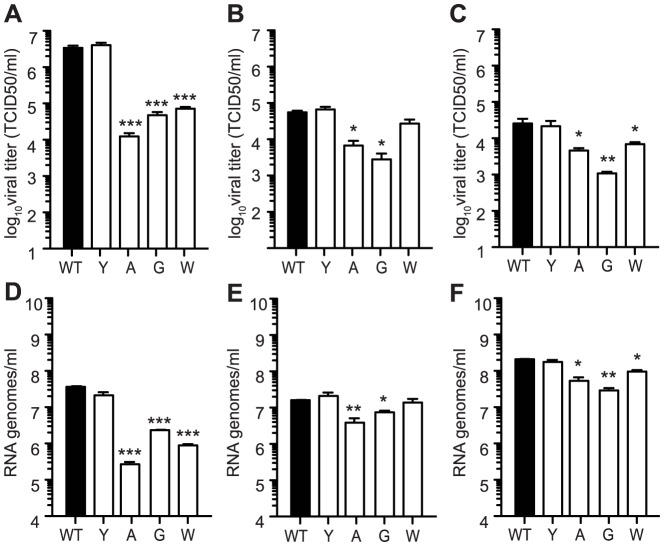
Mutators have replication defects in *Ae. albopictus*- and *Ae. aegypti*- derived cell culture. Production of infectious particles determined by TCID_50_ (A, B, and C) and genome copies determined by qRT-PCR (D, E, and F) were measured at 24 hours by one-step growth kinetics in C6/36 (A and D), Aag2 (B and E), and U4.4 (C and F) cells. While antimutator 483Y is similar to WT CHIKV, all mutators exhibit attenuation in terms of both titer and RNA genomes (mean values ± SEM, n = 3, *P<0.05, **P<0.01, two-way ANOVA with Bonferroni posttest).

Because arboviruses must cycle through both vertebrate and arthropod hosts, and since mutator strains of other RNA viruses were only examined in mammalian systems [6], [7], [19], [24], we addressed viral replication in three mosquito cell lines: *Ae. albopictus* C6/36 cells, *Ae. aegypti* Aag2 cells and *Ae. albopictus* U4.4 cells. The replication profile for the antimutator 483Y was indistinguishable from wildtype in all conditions. On the other hand, the mutator strains 483A, G, and W presented significantly lower infectious progeny in C6/36 (P<0.001 for all, two-way ANOVA; Figure 4A), Aag2 (P<0.05 for 483A and G, two-way ANOVA; Figure 4B) and U4.4 (P<0.05 for 483A and W, P<0.01 for 483G, two-way ANOVA; Figure 4C) cells. Unexpectedly, we observed unprecedented reduction in genomic RNA released into the supernatant in all three mosquito cell types (Figure 4D–F). These results are discordant with the existing literature that found mutator polymerases synthesize RNA at faster rates than wildtype [6], in which case decreases in virus titer resulted directly from the increased mutational burden.

**Figure 5:**
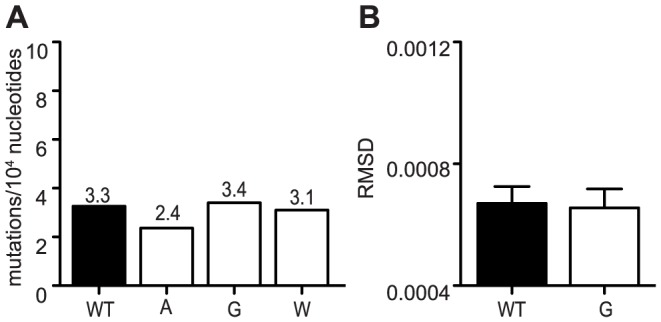
Mutation frequencies of all variants are reduced in *Ae. albopictus* cells. (A) Average mutation frequencies determined by molecular clone sequencing of WT and mutator strains in C6/36. Approximately 75 clones were sequenced per variant. No significant differences were found in mutation frequencies of mutators compared to WT (χ^2^ test). (B) Average diversity of WT and mutator 483G at each position across the genome. There were no significant differences found between WT and 483G. The root-mean-square-deviation (RMSD) is shown (mean values ± SEM, *P<0.05, Mann-Whitney *u* test).

**Figure 6:**
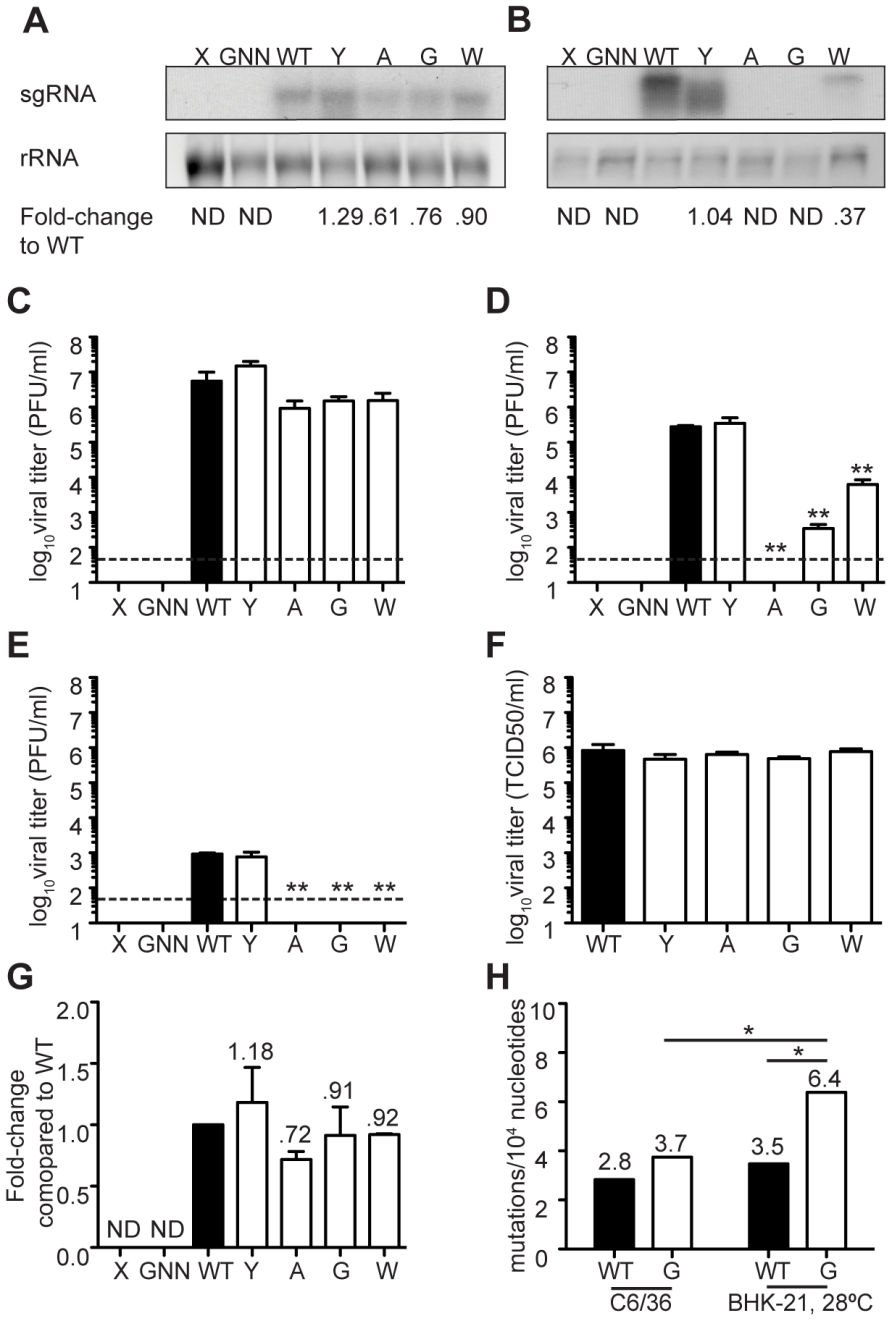
Mutator attenuation in mosquito cells is due to a replication defect. (A) BHK-21 cells or (B) C6/36 cells were transfected, and 48 hours later intracellular RNA was collected for northern blot analysis. (A) In BHK-21 cells, all mutator variants were able to synthesize subgenomic RNA (sgRNA). (B) In C6/36 cells, mutator sgRNA was undetectable or greatly reduced. Loading control shows ribosomal RNA (rRNA). Fold-difference compared to WT was calculated in Image J (n = 2; ND, not detected). Titers of virus progeny recovered from the supernatant from the transfections in (C) BHK-21 cells from A or (D) C6/36 cells from B were determined by plaque assay (mean values ± SEM, n = 2, **P<0.01, one-way ANOVA with Dunn's multiple comparison test; dotted line = limit of detection). (E) U4.4 cells were transfected and 48 hours later viral progeny from the supernatant was titered by plaque assay (mean values ± SEM, n = 2, dotted line = limit of detection). (F) Production of infectious particles determined by TCID_50_ was measured by one-step growth kinetics in BHK-21 cells at 28°C. No significant difference compared to WT was found for any variant at 24 hours (mean values ± SEM, n = 3, Student's *t* test). (G) BHK-21 cells were transfected, virus was grown at 28°C, and 48 hour later intracellular RNA was analyzed by northern blot. Fold-difference compared to WT was calculated in ImageJ (n = 2; ND, not detected). (H) Mutation frequencies were compared between WT and mutator 483G in both C6/36 and BHK-21 cells grown at 28°C.

Here, the reduced viral titers obtained in mosquito cells seem to result from a host-specific replication defect, rather than the effect of lethal mutation. To further distinguish between these two effects, we examined whether mutation frequencies differed in mosquito versus mammalian cells, comparing wildtype CHIKV to the mutator strains. It is important to note that because mutator strains replicate so poorly in mosquito cells, these strains may present artificially low mutation frequencies. Unfortunately, it is not possible to uncouple replication from mutation frequency in this model. Nevertheless, the mutation frequencies of all viruses, including wildtype), were lower in C6/36 cells (Figure 5) than in BHK-21 cells (Figure 1B). Furthermore, the significant differences that existed between mutators and wildtype in mammalian cells were negated in mosquito cells, as evidenced by molecular clone sequencing (Figure 5A) and whole-genome deep sequencing (Figure 5B). We thus hypothesized that the negative fitness cost of mutator polymerases in mosquito cells is more closely linked to replication defects. To further confirm this, we generated genetically homogenous *in vitro* transcribed RNA corresponding to each variant, which do not present the differences in mutation frequencies of virus stocks generated in cell culture. Following transfection of mammalian BHK-21 cells, there were no significant differences in RNA synthesis (Figure 6A) or production of infectious virus (Figure 6C); however, in mosquito C6/36 cells, there was a very marked defect in replication for the mutator variants, compared to wildtype virus or the antimutator 483Y strain (Figure 6B), that correlated with the significant reduction in progeny (P<0.01 for all mutators, one-way ANOVA; Figure 6D). Similarly, no detectable infectious progeny was produced following transfection of U4.4 cells with the mutator variants (Figure 6E), further confirming the replication defect observed during infection of cells with virus stocks.

To exclude the possibility that this replication defect is the result of temperature-sensitivity rather than host-specificity, we performed infections in mammalian BHK-21 cells at 28°C (mosquito cell temperature). We observed no difference in the growth of any variant compared to wildtype (Figure 6F). In addition, we transfected mammalian cells grown at 28°C, and saw no difference in subgenomic RNA synthesis, indicating that the reduced polymerase processivity of mutators in mosquito cells is not due to reduced temperature (Figure 6B and 6G). Finally, we determined whether lower temperature could be responsible for the reduced mutation frequencies we observed in mosquito cells. In mammalian cells at 28°C, mutator 483G makes significantly more mutations than in mosquito cells at 28°C (P<0.05, χ^2^ test; Figure 6H). In contrast to what we observed in mosquito cells, mutator 483G also made significantly more mutations than WT (P<0.05, χ^2^ test; Figure 6H). These data indicate that lower temperature is responsible for neither the replication defects nor the reductions in mutation frequencies we observed in mosquito cells.

### In the mosquito host, selective pressure against the replication defects of mutator strains causes reversion

**Figure 7:**
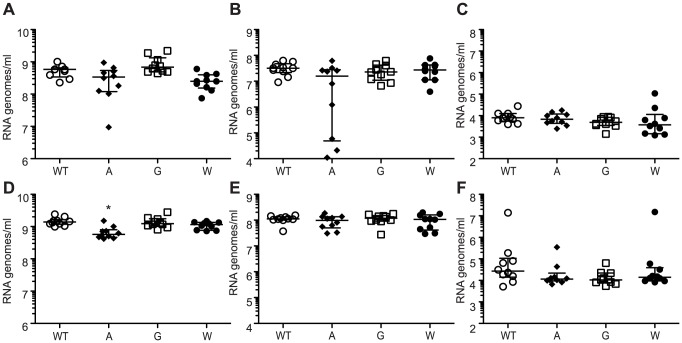
Mutators are under strong pressure to revert in mosquito models. (A–C) *Ae. aegypti* or (D–F) *Ae. albopictus* were infected with WT and mutator strains of CHIKV, and mosquitoes were collected at 1, 3, 7 and 14 days post-infection. *Ae. aegypti* mosquitoes exhibited no significant differences in RNA genomes in (A) bodies, (B) legs and wings, or (C) saliva at 7 days post-infection. Seven days post-infection *Ae. albopictus* mosquitoes exhibited no differences in RNA genomes in (D) bodies with the exception of a significant but slight decrease for 483A. There were no significant differences observed in the (E) legs and wings or (F) saliva (median values ± IQR, n = 10, *P<0.001, two-way ANOVA with Bonferroni posttest).

Since host-specific replication defects were observed in mosquito cell culture, we hypothesized that these variants would be even more attenuated in mosquitoes than in mice. We orally infected both *Aedes* species CHIKV hosts (*Ae. albopictus* and *Ae. aegypti*) with a blood meal containing either wildtype or the 483A, G and W mutators. Seven days after infection, when CHIKV has reached peak titers, we quantified viral loads in bodies (infection), legs and wings (dissemination) and saliva (transmission) of individual mosquitoes (Figure 7). Surprisingly, no significant defect was observed in either *Aedes* species for any of the variants. To address the possibility that the fitness cost of defective replication, observed in mosquito cell culture, would favor the reversion of these mutant polymerases to wildtype, we deep sequenced virus from the body of an individual mosquito that presented the median titer from each group. Indeed, reversion to wildtype (or other replication competent variants, such as 483T or 483V) occurred in 483A (81%), 482G (93%) and 483W (39%). Whether position 483 changed to wildtype depended on the genetic distance of the mutated codon from wildtype: for example, W (TGG) reverted completely to WT (TGT), while A (GCT) reverted predominantly to a combination of V (66%; GTT) and T (ACT; 13%). Interestingly, when we examined higher passages (passage 3) of mutators in C6/36 cell culture, we also observed varying levels of reversion (ranging from less than 1% to as much as 50%), highlighting the strong selective pressure acting against this replication defect.

### Confirmation of the role of residue 482 in Sindbis virus in altering fidelity and affecting virus fitness *in vivo*

**Figure 8:**
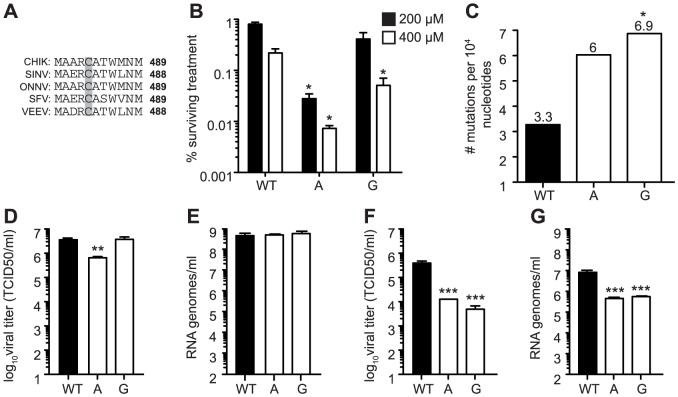
Mutagenizing SINV position C482 generates mutator strains that exhibit replication defects in mosquito cells. (A) Alignment of five alphaviruses at the conserved cysteine residue at position 482/483 (SINV/CHIKV) in CHIKV, SINV, Semliki forest virus (SFV), O'nyong nyong virus (ONNV), and Venezuelan equine encephalitis virus (VEEV). (B) Ribavirin sensitivity of potential SINV mutators. HeLa cells, treated with 200 µM or 400 µM ribavirin, or left untreated, were infected at an MOI of 0.1. The percentage of infectious progeny virus surviving treatment at both concentrations of ribavirin relative to the untreated control is shown (mean values ± SEM, n = 3, *P at least<0.05, two-way ANOVA with Bonferroni posttest). (C) Average mutation frequencies determined by molecular clone sequencing of WT SINV and variants 482A and G. Approximately 75 clones were sequenced per variant (*P<0.05, χ2 test). (D–G) Viral titers determined by TCID_50_ (D and F) and RNA genomes determined by qRT-PCR (E and G) of SINV mutators in (D and E) BHK-21 cells and (F and G) *Ae. albopictus* C6/36 cells. Mutator variants exhibit significant defects in C6/36 but these defects are reduced in BHK-21 cells (mean values ± SEM, n = 3, **P<0.01, ***P<0.001, two-way ANOVA with Bonferroni posttest).

After confirming that polymerase position 483 plays an important role in modulating fidelity in CHIKV, we examined if this residue is a universal fidelity determinant among the alphaviruses. Indeed, this region of the nsp4 gene containing a cysteine is conserved across the alphavirus family (Figure 8A). Thus, we generated the analogous fidelity variants (482A, G, W) in the well-studied, distantly related alphavirus Sindbis virus (SINV). Genetically stable mutants (482A and G) were screened for changes in ribavirin sensitivity. Both showed significantly higher sensitivity than wildtype SINV (for 482A, at least P<0.01, for 482G, P<0.05, two-way ANOVA; Figure 8B). Moreover, the mutation frequencies determined by molecular clone sequencing confirmed the mutator phenotypes suggested by ribavirin screening (Figure 8C): in comparison to wildtype that presented 3.3 mutations per 10,000 nucleotides, 482A presented 6.0, and 482G presented 6.9 (P<0.05, χ^2^ test). This confirms that this conserved residue is a general fidelity determinant for the alphaviruses.

**Figure 9:**
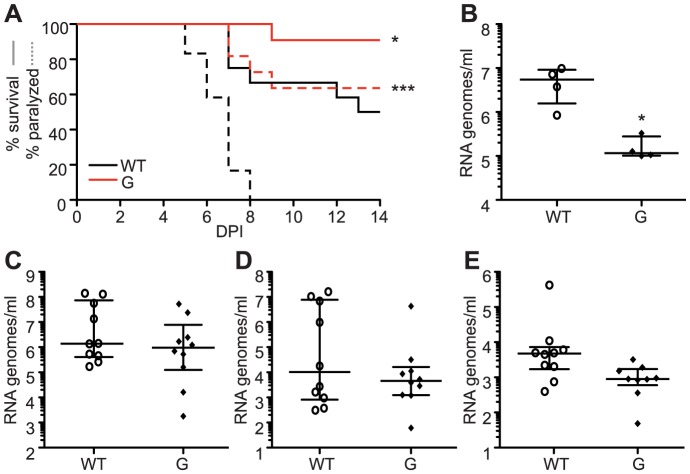
SINV mutator 482G is attenuated in mice but reverts in mosquitoes. (A) Percent survival (solid line) is significantly increased and paralysis (dashed line) is significantly reduced in mice inoculated with mutator 482G compared to WT (*P<0.05, ***P<0.0001, χ^2^ test). (B) Seven days post-infection, mutator 482G has significantly fewer RNA genomes present in the brain of mice than WT (median values ± IQR are shown, n = 4, *P<0.05, Student's *t*-test). DPI, days post-nfection. (C–E) Mutator 482G reverts in mosquitoes, exhibiting similar amounts of viral genomes as WT in the (C) bodies, (D) legs and wings, and (E) saliva (median values ± IQR, n = 10, Student's *t*-test).

We next addressed whether replication defects also existed for these SINV mutators. In mammalian BHK-21 cells, mutator variants produce near wildtype-like titers of infectious particles (Figure 8D), and the same amounts of extracellular RNA genomes (Figure 8E). Importantly, as was observed for CHIKV strains, the SINV mutators presented more significant drops in virus titers in mosquito C6/36 cells (P<0.001, two-way ANOVA; Figure 8F), that correlated with a significant decrease in extracellular RNA genomes (P<0.001, two-way ANOVA; Figure 8G). Given the similarity of *in vitro*, host-specific phenotypes of CHIKV and SINV mutators, we hypothesized that SINV mutators would behave as CHIKV mutators *in vivo* (exhibiting attenuation in a mouse model and reversion in mosquitoes). We inoculated 8-day old mice with wildtype and 482G SINV strains, and observed significantly higher survival in mice infected with the mutator (91% compared to 50% for the wildtype, P = 0.0474; Figure 9A). In addition, only 36% of mice inoculated with 482G exhibited complete hind limb paralysis, compared to 100% of mice infected with wildtype SINV (P<0.0001, χ^2^ test; Figure 9A). Interestingly, this reduced paralysis correlated with significantly lower titers in the brain at day 7 post-infection (P<0.05, Student's *t*-test), confirming the attenuation of mutator strains in mammalian models in yet another virus (Figure 9B). We next examined the *in vivo* phenotype of SINV mutator 482G in *Ae. aegypti* mosquitoes. As expected, we observed reversion of position 482G to wildtype, and therefore, no differences in titers in the mosquito host (Figure 9C–E).

**Figure 10:**
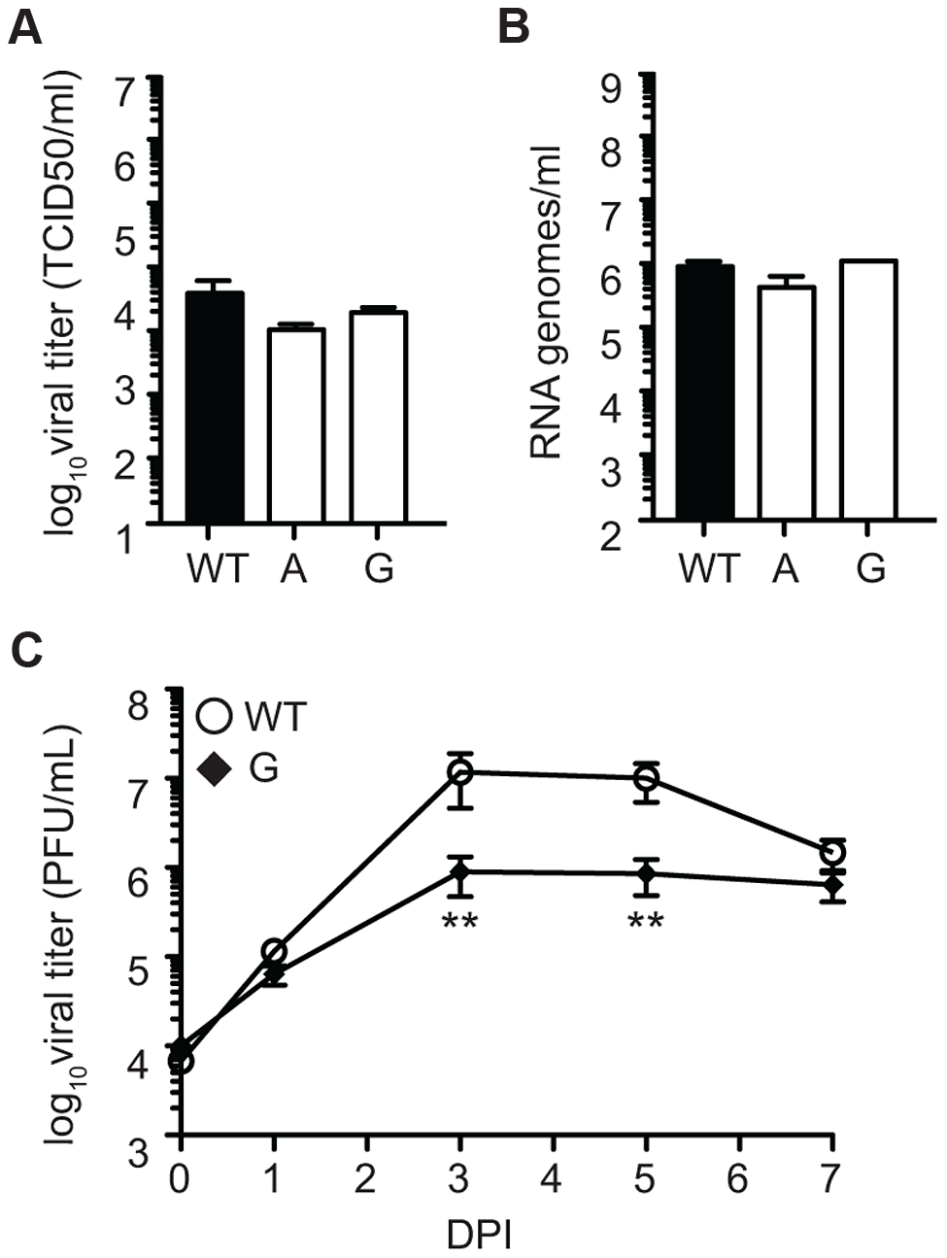
SINV mutator 482G is attenuated in fruit flies. Viral titers determined by TCID_50_ (A) and RNA genomes determined by qRT-PCR (B) in *D. melanogaster* S2 cells. No significant differences are found in replication of mutator 482G compared to WT (mean values ± SEM, n = 3, two-way ANOVA with Bonferroni posttest). (C) Fruit flies were injected with either WT or mutator 482G and titers were monitored by plaque assay over the course of seven days. At days three and five, mutator 482G had significantly lower titers than WT (mean values ± SEM, n = 9, *P<0.05, Student's *t* test). DPI, days post-infection.

Since SINV has a broader host range than CHIKV, we examined whether the replication defect was mosquito cell-specific, or more general to insects, by infecting *Drosophila* S2 cells. Interestingly, the mutator strains were replication competent, generating virus titers (Figure 10A) and RNA genome copies (Figure 10B) at levels comparable to wildtype virus. Finally, we injected *Drosophila* with SINV wildtype and 482G mutator and followed the kinetics of infection by titering virus in flies for seven days post-infection. In contrast to CHIKV and SINV mutators in mosquitoes, when *Drosophila* flies were infected with wildtype and mutator strains of SINV, mutator 482G presented significantly lower titers than wildtype on day 3 and 5 (P<0.01, Student's *t*-test; Figure 10C). Sequencing of virus from 482G-infected flies at day 3 and 5 confirmed that no reversion had occurred. These results indicate that in principle, mutators can be attenuated in insects.

## Discussion

Previous work on antimutator CHIKV 483Y suggested this residue could be important for determining intrinsic RdRp fidelity [25]. Although there is no crystal structure available for an alphavirus RdRp, structural models predict that position 483 is located in the same area of the RdRp that generated Coxsackie virus RdRp fidelity variants (Figure S1) [6]. By substituting all other amino acids at this position, more mutator variants were isolated than antimutators, consistent with variants obtained for Coxsackie virus and with variants identified by characterizing the mutation frequencies of previously published reverse transcriptase variants for HIV [6], [32]. To date, all viable RdRp fidelity variants present error rates that remain within the same order of magnitude as their wildtype counterpart [5], [6], [14], [25], [71], [72]. Interestingly, although biochemical assays using purified RdRp of picornaviruses indicate that altering fidelity beyond an order of magnitude is enzymatically possible, these viruses are not viable [73]. Together, these studies suggest that within this viable range, wildtype fidelity sits closer to higher fidelity than lower fidelity. This may be further reflection of how RNA viruses are considered to exist close to a maximum threshold of error [74]. In support of this, in conditions where this reversion does not occur, mutator CHIKV variants present more significant fitness defects *in vivo* (Figure 3) than antimutator virus [25].

A review of the antimutator and mutator RdRp variant literature in virology reveals the following trends: antimutator strains tend to generate less RNA *in vitro*, but have higher specific infectivity, and have only been reported to lose fitness *in vivo* or in competition assays (Figures 2 and 4, and [16], [19], [25]); while mutators generate more RNA *in vitro*, but of lower specific infectivity, with more prominent fitness defects in mice [6]. Accordingly, CHIKV mutators showed congruous trends, exhibiting no significant replication defects in BHK-21 cells, but showing marked attenuation in the mouse model. Importantly, we showed that the mutator status of these variants (higher mutation frequencies) was maintained in the mouse model at the primary site of CHIKV replication and was likely responsible for the observed attenuation (Figure 3).

However, when we examined CHIKV mutators in the invertebrate host, the previous trends for how mutators behave was reversed. First, the differences in mutation frequencies between wildtype and mutator strains became virtually indistinguishable in mosquito cells (Figure 5), although it is difficult to draw clear-cut conclusions given the reduced replication rate of mutators. For all viruses, mutation frequency was lower in mosquito cells compared to mammalian cells (Figure 1). The role that these differences may play in arbovirus evolvability and fitness remain contradictory. Our observations corroborate previous observations in alphaviruses that inter-host cycling slows adaptation [33], [35], [75]; while flavivirus studies report that diversity is maintained in the mosquito host [38], [41], [76], [77], [78], [79], [80]. Second, and contrary to expectations, we observed a severe replication defect in three different mosquito cell cultures (Figure 4), which had never been observed for RdRp fidelity variants in mammalian cell culture. The lower titers of infectious progeny were not the result of accumulation of detrimental mutations as was observed for mutators in mammalian hosts; rather, there was a direct defect in genomic RNA synthesis in mosquito cells (Figure 4 and 6). Interestingly, similar host-specific replication defects were observed for RdRp mutants of West Nile virus (although it is unclear if these variants have altered fidelity). While differences in host temperature do not seem to be the cause, the cellular host factors implicated or missing in these host cell lines remain to be elucidated. Finally, we could not address whether mutators were attenuated *in vivo* in mosquitoes; sequencing of virus populations from mosquitoes revealed partial or total reversion of the fidelity-altering residues at position 483. Although one could expect a variant with severe replication defects to be highly attenuated, it is possible that when coupled to a mutator phenotype, reversion would more quickly and favorably occur when the pressure to increase replication remains, as is the case in mosquitoes that are persistently infected. Whether this defect is general to all mutators in mosquitoes, or whether only amino acids A, G, and W at position 483 bear this curiously coupled mutator/replication effect remains to be seen (since not all variants at this position were defective, as 483Y has no replication defects in mosquito cells [25]). Isolation of additional arbovirus mutators mapping to other residues in the polymerase should resolve this issue.

Since the cysteine at position 483 is conserved in the alphavirus genus, we obtained additional arbovirus mutators in Sindbis virus. SINV mutators also showed severe replication defects in mosquito cells, and SINV mutator 482G exhibited the same phenotypes we previously observed in CHIKV mutators in both mice and mosquitoes. However, the wider host range of SINV allowed us to test whether these replication defects occur across all insects or if they were mosquito-specific [81], [82]. In S2 cells, mutators did not present replication defects, allowing us to test, in principle, whether mutators could be attenuated in an insect model (*Drosophila* flies). Indeed, in the absence of any *in vitro* replication defect and resulting pressure to revert, the mutator strain was attenuated in fruit flies. Thus, our results confirm that arbovirus mutators can, in principle, be attenuated in insects.

Since the isolation of the first antimutator variant of a RNA virus, the growing body of literature shows that either increasing or decreasing replication fidelity has detrimental effects to virus fitness [6], [7], [16], [19], [24], [25]. However, how mutation rates and replication capacity are coupled will require more study, and the degree of attenuation resulting from altering these biochemical properties needs to be more carefully evaluated. A future challenge will be to quantitatively link the measurements of mutation frequencies (average mutations per nucleotide sequenced) performed in this work to actual mutation rates (average mutations per nucleotide site per replication) [2], [83] and to *in vitro* biochemical fidelity (rates of incorporation of correct and incorrect nucleotides in absence of selection)[6], [8], [73], [84], [85], [86], [87]. It is possible that the higher mutation frequencies measured for these alphavirus mutator strains are partly skewed by their producing more RNA genomes in shorter replication cycles and thus accumulating mutations more rapidly, rather than incorporating more errors per genome during each replication cycle. Indeed, biochemical studies of single-nucleotide incorporation by other mutator polymerases confirm that mutators are both faster enzymes and have higher frequency of mis-incorporation events per replication. In absence of a biochemical assay for alphaviruses, new technologies using microfluidic single-cell analysis of virus strains during single replication cycles should help correlate mutation frequencies, mutation rates, and enzyme fidelity with more confidence.

Recent studies have proposed both antimutator and mutator strains as candidates for rationally designed live attenuated vaccines [6], [7], [19], [25], [71]. Overall, fidelity variants present attenuated titers *in vivo* that range from one to several orders of magnitude lower than wildtype virus. Whether this degree of attenuation is sufficient to elicit protective immunity without causing disease will require more careful evaluation in more relevant animal models, as virtually all work has been performed in mice using viruses that are often not natural mouse pathogens. *In vitro* systems and artificial hosts may alter many of the selective pressures to which a virus would be subjected in a natural host [88], [89], [90], [91]. The present study and other work highlight that intrinsic fidelity and the mutant spectrum are labile and subject to stringent and disparate selective pressures in different hosts [34], [35], [75], [76], [79], [82], [92], [93]. A more comprehensive understanding of the selective pressures in natural hosts is crucial to predicting how viruses will behave *in vivo*, and essential to evaluating the feasibility of using fidelity variants as vaccines, whether stand-alone or coupled with other, conventional attenuating mutations. Despite the necessity for further research, from a vaccine development perspective these data support that in principle, mutators can be attenuated in a wider range of hosts and may be viable candidates for live-attenuated vaccines.

## Supporting Information

Figure S1 **Structural homology model of the CHIK nsp4 core polymerase.** The model shows the predicted locations of C483 (green sphere) and two nearby residues (L368 and T370, shown as gold spheres) that are the structural equivalents of known fidelity-altering sites in Coxsackie virus polymerase (positions I230 and F232, respectively) (6). The model was obtained using the I-TASSER threading platform (Roy, A., et al., 2010) and is color coded according to polymerase domains. The polymerase palm domain (grey), where our fidelity altering mutations are located, is modeled with fairly high confidence because of the large number of conserved polymerase sequence motifs (motifs A–D) whose structure is also well conserved among the solved RdRp structures. The thumb domain (purple) modeling is less reliable, but secondary structure prediction of the nsp4 sequences is wholly consistent with the alpha-helix based structure of this domain in known RdRP structures. Finally, modeling of the fingers (red) domain is the least reliable as a result of significant sequence and length divergence in this region of RdRPs. Domains where the modeling is weak are shown as semi-transparent.
(TIF)
