# Supplementary figures and images for: Alphavirus Mutator Variants Present Host-Specific Defects and Attenuation in Mammalian and Insect Models
Source: PLoS Pathog. 2014 Jan 16;10(1):e1003877. doi: 10.1371/journal.ppat.1003877 (PMC3894214; doi:10.1371/journal.ppat.1003877)

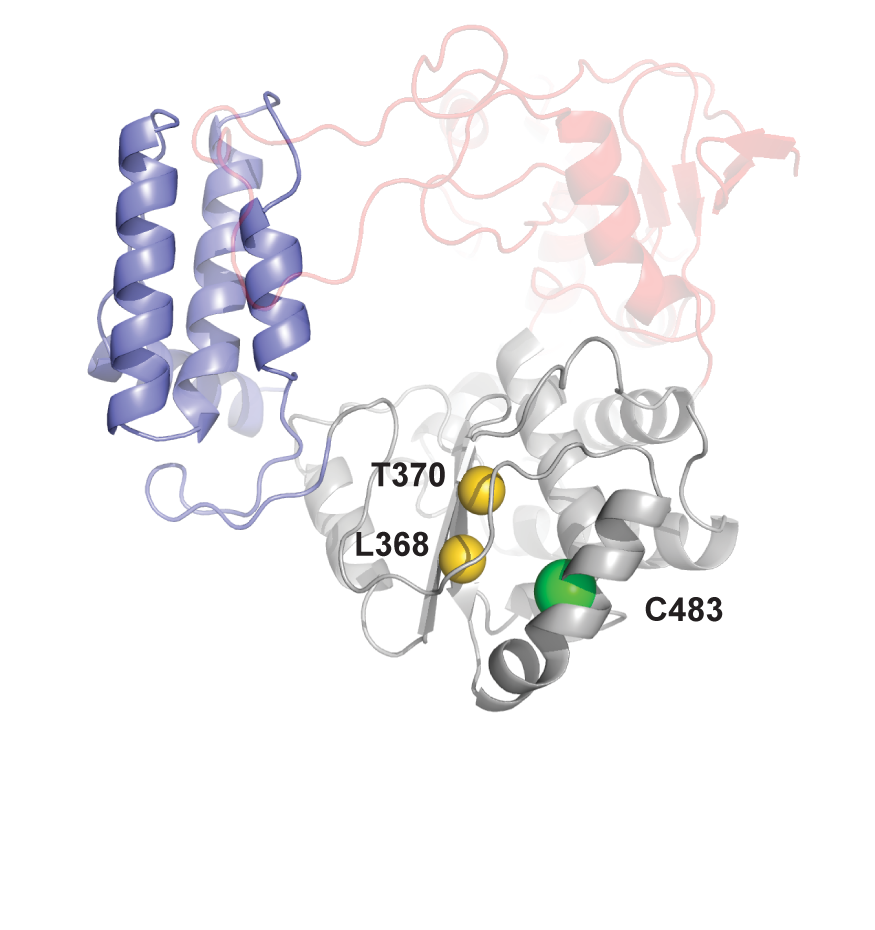

Supplement: Figure S1 — Structural homology model of the CHIK nsp4 core polymerase. The model shows the predicted locations of C483 (green sphere) and two nearby residues (L368 and T370, shown as gold spheres) that are the structural equivalents of known fidelity-altering sites in Coxsackie virus polymerase (positions I230 and F232, respectively) (6). The model was obtained using the I-TASSER threading platform (Roy, A., et al., 2010) and is color coded according to polymerase domains. The polymerase palm domain (grey), where our fidelity altering mutations are located, is modeled with fairly high confidence because of the large number of conserved polymerase sequence motifs (motifs A–D) whose structure is also well conserved among the solved RdRp structures. The thumb domain (purple) modeling is less reliable, but secondary structure prediction of the nsp4 sequences is wholly consistent with the alpha-helix based structure of this domain in known RdRP structures. Finally, modeling of the fingers (red) domain is the least reliable as a result of significant sequence and length divergence in this region of RdRPs. Domains where the modeling is weak are shown as semi-transparent. (TIF) [file ppat.1003877.s001.tif]
